# Evaluating a Codesign Process in Mental Health: ‘Harnessing the Power of Together’

**DOI:** 10.1111/hex.70371

**Published:** 2025-08-10

**Authors:** Michelle Kehoe, Hannah Friebel, Kirsty Rosie, Paul Kremer, Frances Shawyer, Graham Meadows, Ingrid Ozols

**Affiliations:** ^1^ Department of Occupational Therapy Monash University Melbourne Australia; ^2^ Department of Psychiatry Monash University Monash University Australia; ^3^ Southern Synergy, Department of Psychiatry Monash University Melbourne Australia; ^4^ Monash Health Melbourne Australia; ^5^ Mental Health Program, Monash Health Melbourne Australia; ^6^ School of Primary and Allied HealthCare Monash University Melbourne Australia; ^7^ Monash Centre for Health Research and Implementation, Faculty of Medicine, Nursing and Health Sciences Monash University Melbourne Australia; ^8^ Centre for Mental Health and Community Wellbeing, School of Population and Global Health University of Melbourne Melbourne Australia

**Keywords:** carer, codesign, consumer, education, frameworks, mental health, power, restorative

## Abstract

**Background:**

Mental health services often undertake codesign processes to inform and enhance service delivery, however, there continue to be many challenges undertaking codesign activities in mental health.

**Objective:**

The aim of this study was to evaluate a codesign process undertaken for a project seeking to enhance consumer choice and satisfaction at one acute mental health inpatient setting in Melbourne, Victoria.

**Method:**

A five‐stage codesign process evaluation was undertaken. Data collected at each stage included surveys, feedback and reflections. The data was analysed using basic data analysis and a thematic approach. Data was used for the evaluation of each stage and to inform subsequent stages. This paper describes the various activities undertaken within the codesign process which we sought to examine post hoc using the context, input, process and product (CIPP) framework.

**Findings:**

The findings derived from the various data collection stages including the workshops and staff reflections highlighted two main foci, ‘direct experience’ and ‘the bigger picture’. First, the participants from the codesign workshops highlighted their experience including both positive experiences such as feeling heard and seen, and challenges such as having a lack of knowledge. For staff, the focus was on the bigger picture around codesign such as engaging consumers and adapting the approach as needed.

**Discussion:**

The study highlighted some of the challenges and benefits found undertaking a codesign process. In particular engagement with stakeholders required a higher level of open communication before workshops being undertaken which was important to address a power imbalance. However, the results were limited due to a lack of consumer and staff feedback. The team reflections sought to provide reasons for this limitation which was attributed to a lack of organisational readiness, stakeholder engagement, differing expectations, lack of time, and power differentials. The CIPP framework was used as a tool to undertake a post hoc evaluation of this complex codesign process undertaken.

**Conclusion:**

Codesign processes will continue to grow as the preferred method of ensuring mental health services meet the needs to the community. The CIPP framework is one means of ensuring that codesign processes follow a systematic, iterative approach to appropriately meet those needs. Future projects within mental health settings should consider the inclusion of consumers, carers and non‐lived experience staff members, as a project team, to offer differing views. In addition, project success often relies on organisational readiness and the ability to reflect on and address power. We conclude that a restorative practice approach to create more open dialogue and communication before commencement of codesign projects may be a key solution.

**Patient and Public Contribution:**

The authors would like to thank and acknowledge the consumers with a lived experience, carers, and mental health care staff who participated in the codesign process. This paper was written by consumers, carers, staff and academics who were involved in the project.

## 1 Introduction

Codesign and coproduction are becoming increasingly valued in the development, design, and improvement of mental health services [1, 2, 3]. In the past many mental health services took a top‐down approach whereby staff with clinical experience were considered the experts throughout this process [3, 4]. However, in more recent years there has been growing recognition that those with mental health conditions are the experts of their own experience [5]. This concept is arguably at the heart of the change from the top‐down approach to that of empowering consumers. Codesign is one way to include the consumer voice in service improvement often using multiple stages and methods such as surveys, interviews and/or workshops [6]. Despite its increasingly high profile, codesign remains confusing to many and has many challenges. To illustrate and better understand the nuances involved in conducting codesign, the current study reports on a codesign process undertaken for a project aimed at improving treatment choices, satisfaction, and recovery for consumers within in an acute mental health setting in Melbourne, Victoria.

### 1.1 Codesign and Coproduction Terminology

Although the term codesign is often used interchangeably with coproduction, these two concepts are distinctly different. Codesign is one component within a coproduction process, coproduction being a methodology or philosophy that incorporates brainstorming (sometimes called cocreation), planning, design, development, implementation, and evaluation [2]. Although many claim to be following a coproduction process this is rarely the case due to the complex and time‐consuming nature of involving a wide range of stakeholders at every stage of a project [7, 8, 9]. The issue of time is often underestimated since it can be a lengthy process to ensure representation, establish rapport and overcome any barriers to engagement [8, 10]. A further challenge is a lack of common terminology or process resulting in “fuzzy terminology” (p. 54) [4, 11]. This can result in a lack of consistency and understanding around what constitutes codesign or coproduction. In simple terms codesign, for the current project, is service improvement design *with* people rather than *for* people.

### 1.2 Practical Challenges and Benefits

It has been widely reported that involvement of consumers in service reform including design, development, implementation and delivery comes with challenges [3]. For consumers, there may be a reluctance to be involved due to prior negative experiences within the mental health service which may have resulted in a lack of autonomy [12]. There has also been criticism that consumer involvement is often tokenistic involving those who are less likely to upset the status quo [13]. This tokenistic approach can be attributed, in part, to a reduction in consumer power by placing the consumer at the bottom of the hierarchy. A power imbalance can occur when the agenda, and rules associated with the codesign process only involve those in decision‐making positions [14]. This can result in consumers lacking validation and feeling unheard and disempowered [4, 15]. Further issues with the codesign can be due to a lack of understanding within a messy process. However, there is a paucity in literature which explicitly discusses the need to upskill or train people either with or without a lived‐experience around aspects of codesign. This issue was highlighted in a recent Delphi study by Krynsinska and colleagues [16] who concluded that training for researchers and people with a lived‐experience is a key aspect to successful coproduction.

To be authentic, it is critical that codesign allows adequate time to listen and understand various viewpoints. This can be a challenge when the participant or communities are not ready to move forward and yet the codesign facilitators feel pressured due to time constraints [10]. Although there has been some progress in addressing some of the issues, the process of codesign continues to mystify and perplex many seeking to undertake and understand the value of the process.

Despite the challenges of codesign there can be multiple benefits for those involved in the codesign process [2]. When consumers are engaged in the design and development of programmes and interventions it can result in a better understanding of user needs and in acceptability [17]. In addition, the codesign process can enhance skills, knowledge, confidence and a sense of empowerment for those involved [2]. A recent codesign study by Kehoe and colleagues [9] found participants felt a genuine contribution to “making a difference in the lives of others” (p. 7) and participation enabled the ability to “see the big picture” (p. 7). However, one key aspect to ensure success of a codesign process is the need to tailor the approach to the specific context [6].

In the current study, the authors use Stufflebeam's Context, Input, Process and Product (CIPP) [18] framework as a post hoc tool to help structure the evaluation of a codesign process following completion. This paper will describe the actual stages of the codesign process undertaken, the alignment with the CIPP framework, and the results of each component. Consideration will be given to how well the actual stages of the project align with the CIPP framework. In the first instance a brief overview of the CIPP framework is described followed by a description of the “consumer choice” project that underwent this co‐designed process.

### 1.3 The CIPP Framework

**Figure 1:**
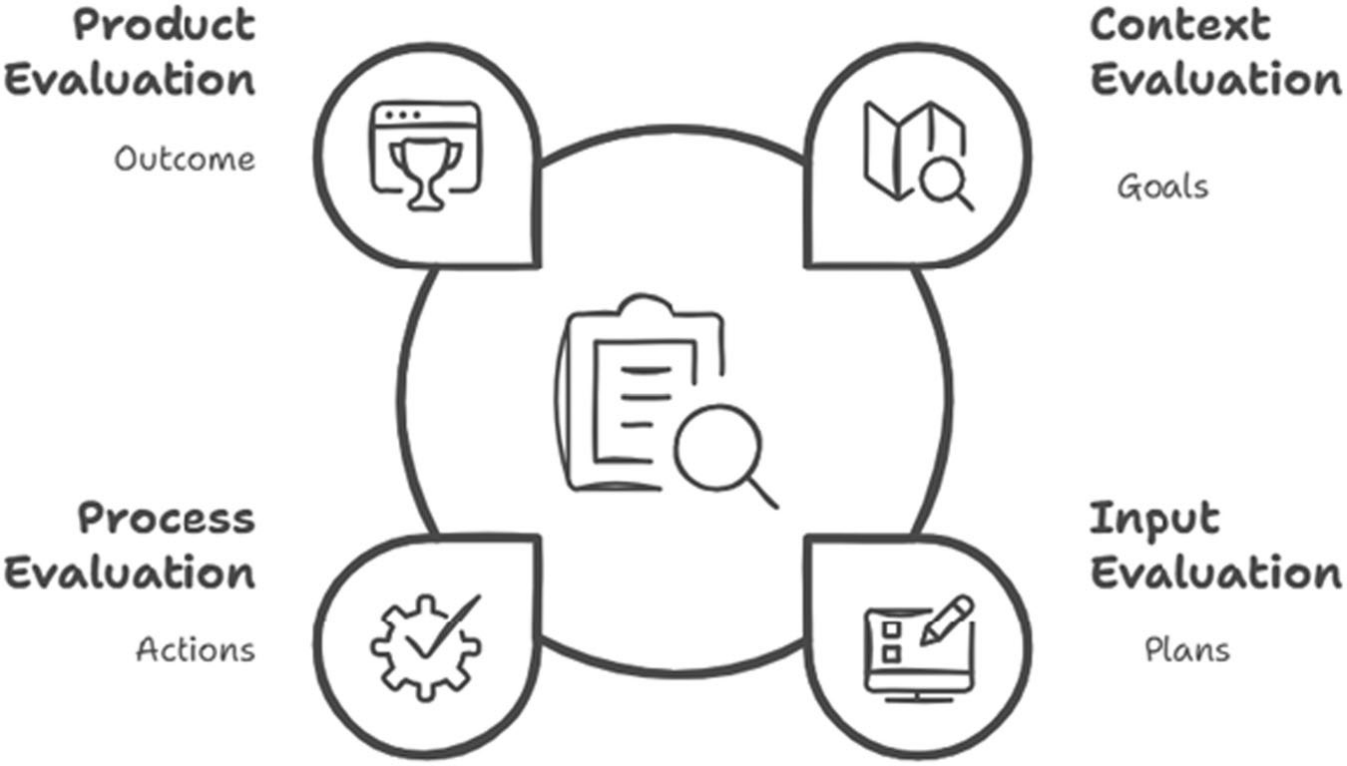
Adapted version of Stufflebeam (2015) CIPP framework.

The CIPP framework by Stufflebeam [18] was designed to provide a systematic and comprehensive framework for evaluating programmes, projects, institutions (e.g., schools), and systems. In practice, the CIPP framework is a checklist enabling evaluators to provide both formative and summative reporting on the progress of projects over time. The first aspect of the CIPP framework, *context*, seeks to assess needs, problems and opportunities, to address the question, ‘what needs to be done?’. The *Input* component examines strategies, resources, and action plans, addressing the question ‘how should it be done?’. The *process* component, monitors the plans and activities responding to the question ‘Is it being done?’. Finally the *product* component examines the effectiveness of outcomes and impacts, addressing the question ‘Is it succeeding?’ [19]. Figure 1 shows the interactions between the evaluation components of the CIPP framework, the main aims with the core values being at the centre.

The CIPP framework is designed to systematically consider all components of a project including commencement, during the process iteratively, and at the implementation stage with an emphasis on learning by doing [20]. Specifically, the framework aims to assist stakeholders to identify needs within a community, seek a response to address those needs, monitor the process and any potential barriers, thereby making any adjustments as needed, and finally judging the merit of the outcomes from the project [20]. Because these aims are congruent with those of codesign, we chose to use the CIPP framework post hoc to determine how well the steps undertaken in our codesign process align with the framework as part of the evaluation.

### 1.4 The Consumer Choice Project

The consumer choice project that was co‐designed in this study was prompted following the recommendations of the Royal Commission into Victoria's Mental Health system [21]. Its purpose was to enhance consumer choice to improve satisfaction and recovery outcomes in an acute mental health inpatient setting in south‐east Melbourne, Victoria. The project team was established to identify, through codesign, possible treatment support options which could improve the acute mental health in‐patient experience and subsequently implement the recommendations from the various codesign phases. The specific recommendations emanating from the codesign and their implementation is not a focus of the current paper since the focus is on the codesign process and activities. In alignment with prior research, the project sought to undertake a customised approach to account for the context and cohort [8]. The project was led by lived‐experience experts including a consumer project officer, two consumer project staff and supported by a consultant consumer academic (with both lived‐experience and academic qualifications). In addition, a project implementation group provided a range of perspectives as recommended by McKercher [22]. This included the operations manager, a consumer peer, a carer peer, research academics, and clinical staff. Due to the level of importance placed on quick implementation of the recommendations from the Victorian Royal Commission into Mental Health, there was significant pressure on the project team for a timely codesign process. Despite this, the team chose to follow prior recommendations and ensure that adequate time was provided to build rapport, trust and engagement with key stakeholders [8, 10]. Using the CIPP framework (and checklist) [18], the aim of the current study was to evaluate the codesign process, post hoc, for the consumer choice project to better understand and learn from the challenges and benefits.

## 2 Method

The method section describes the overarching project, the five stages of the codesign including data collection, purpose of the data collection and analysis at each stage. Ethics approval was received from Monash Health and Monash University and was classified as a quality improvement project.

### 2.1 Participants

**Figure 2:**
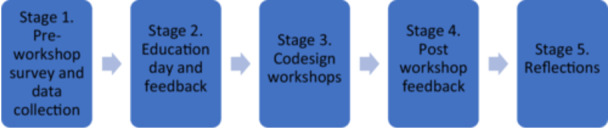
Stages of the codesign process.

All mental health staff (including peer workers) who were either currently or had previously worked in the hospital mental health inpatient unit where the project was based were invited to participate in the codesign process. This was purposefully decided since the project was seeking to implement changes in the same particular acute inpatient unit. Consumers and carers who had experienced a prior inpatient stay within the same mental health unit were also invited to participate. Consent for survey data was implied via completion of the survey. Workshop participants were provided with a verbal explanatory statement about data use during initial engagement with them. Consent was implied if participants chose to opt into attending the workshops. The entire codesign process consisted of five stages which are depicted in Figure 2.

### 2.2 Procedure

The following section outlines the procedure undertaken at each stage of the codesign process.

### 2.3 Stage 1. Pre‐Workshop Survey and Data

The first stage of the codesign process was to explore some initial thoughts on what constituted ‘consumer choice’ in an acute mental health inpatient setting to shape the questions posed during the codesign workshops. The aim was to develop understanding of the current context, for example what types of treatments were currently available to consumers within the acute inpatient setting. Due to COVID‐19 restrictions at the time of the project, an online survey was deemed as the most appropriate means to gather this data. Surveys were distributed via email to all staff current or prior staff who worked in the specific mental health unit. Consumers and carers who had accessed the unit were identified via the peer workforce. Following verbal agreement between the peer and consumer or carer the project team followed up with the email survey.

The survey consisted of a list of supports that were already offered, for example, peer support, and a list of supports that were currently not available but could be potentially helpful if offered, for example animal assisted therapy. Participants were asked to identify supports that they thought were helpful or potentially helpful. In addition, consumers, were asked to provide some additional data including basic demographic information such as (1) duration of stay; (2) number of stays; (3) involuntary treatment status; and (4) provision of an advance care statement. This was followed by three open‐ended questions: ‐

1. What are the most important things for you to have choice and control in an inpatient setting?
2. What if anything is preventing you from having choice?
3. What would support you in having more choice and control in an inpatient setting?

From the 35 people who responded, there were 28 consumers, four carers and three mental health care staff. The data was examined using frequency percentages and the qualitative feedback was thematically analysed [20] (See findings section). Data was collated and presented to participants at the first codesign workshop.

### 2.4 Stage 2. Education Half‐Day Session

The second stage of the project involved an education session which occurred before the workshops. This was determined following the initial survey data collection and through multiple conversations with staff, consumers and carers. It became evident that many of those intending to participate in the workshops were unfamiliar with the codesign process and terminology. As such, the project lead engaged staff from an external local mental health education provider to conduct a pilot half‐day education session to inform participants about coproduction and codesign. All those who had agreed to participate in the codesign workshops were invited to the online education session. Invitations were sent via email and the session was conducted via Zoom. Two independent educators conducted a 3½ hour session with 13 participants (5 staff, 6 carers and 2 consumers). Topics included the philosophy of coproduction, what codesign aims to achieve, how to address any power imbalance, and how participants could gain the most from their participation. A post‐session survey was distributed afterwards to gauge satisfaction with the content.

### 2.5 Stage 3. Codesign Workshops

The codesign workshops were conducted to brainstorm ideas which would improve treatment choices, satisfaction, and recovery for consumers. Because this occurred during COVID‐19 restrictions there were restrictions on how these could be conducted given the project included involvement of staff working in a public health setting. Despite this, it was decided by the project team it would be beneficial in conducting a short face‐to‐face workshop with a small number of consumers to build rapport and to create a safe welcoming environment. The consumer only workshop was attended by five consumers with two lived‐experience project staff. The number of people in attendance was determined by the maximum COVID‐19 room density restrictions at the time. At the commencement of the session a background on the project was provided along with the findings from the survey data collected at stage 1.

Following the consumer only session, three online workshops were conducted with a mix of staff, consumers and carers. The online workshops were 2.5 h duration conducted on zoom using MiroBoard, an online whiteboard style interactive tool to capture participant responses. The first session commenced with a discussion around how to make each workshop a safe and inclusive space to share thoughts and ideas. The Miroboard with the group agreement produced can be found at Appendix B. The group agreement was revisited at subsequent workshops with participants having the ability to change or add any thoughts or ideas. Note: the colours on the Miroboard have no bearing on the content and participants had the ability to choose the colour which they feel represents themselves. Following the establishment of the group agreement and the overview of the survey, which was provided in session one, each workshop followed a similar pattern.

Sessions commenced with all the participants in one ‘zoom‐room’ with the facilitator providing an overview of that session and the input being sought (see Table 1). Once the overview had been provided, each group (consumer, carer or staff) was allocated a ‘room’ to break‐out into discussions. Each room had a host (consumer, carer or staff member, respectively) to capture the discussion and prompt participants with questions. Participants could ‘post’ a sticky note on the board to capture their thoughts or if they were unable to navigate the technology the host could do this for them (see Appendix C for an example). At the end of the discussion participants returned to the main zoom room to share their thoughts. The attendees and agenda for each workshop is outlined in Table 1.

**Table 1:** Workshop information.

Workshop date	Participants	Agenda/Questions
June 2022	19 (7 staff, 1 peer worker, 3 consumers and 8 carers'	Group agreement Overview of survey data What do we need to add to the challenges around choice? What is happening that is already useful? What resources, activities or other key components do we need to improve consumer choice? How can we narrow this down?
July 2022	18 (4 staff, 2 peer workers, 3 consumers, 9 carers)	What clinical service/therapies would be useful to improve diverse consumer choice? What training can we introduce to build staff capacity? How can we support consumer choice through digital provision?
August 2022	14 (3 staff, 5 consumers, 6 carers	How can we help introduce and educate a consumer about the options/choices they have on a ward? What should the orientation process look like? How do we support choice in the community? How do we measure success?

### 2.6 Stage 4 – Postworkshop Feedback

All those who participated in the codesign workshops were eligible to participate in the post workshop feedback. Nine people provided feedback about the codesign workshops. There was one consumer, six carers and one staff member. Two people attended one workshop, four attended two workshops and three attended all three workshops. The data collection involved four quantitative measures and three open‐ended qualitative questions. The quantitative measures asked participants to rate the extent they felt heard, seen, understood and had a voice. Rating from 1 = very much agree to 5 = do not agree. Percentages were calculated for the four categorical questions. The qualitative results were summarised using thematic analysis as recommended by Braun and Clarke [20].

## 3 Findings

The findings described constitute the data from each stage of the project, as described in the method section. Within the results section the relevant elements of the CIPP framework are identified and discussed with the alignment with the stages of the codesign undertaken. A comparison between the stages of the codesign process and the CIPP framework can be found in Appendix Table A1.

### 3.1 Stage 1. Preworkshop Survey (Context)

The results of the pre‐workshop survey provided context and understanding of the current environment with respect to consumer choice in one acute inpatient setting, with the aim of providing broader information to workshop participants. Forty‐two percent of the consumer respondents (*n* = 28) reported five or more inpatient hospitalisations thus indicating that they were familiar with the choices available within the specific inpatient setting. Most (54%) identified that a peer worker was the most helpful support they had received, followed by mental health nurse (50%). Qualitative responses indicated a variety of responses with respect to what was important for choice in an inpatient setting, what was preventing choice and what supported choice. There were four main, overlapping themes which included diversity of choice (e.g., treatment), training to build staff capacity (e.g., more effective communication), digital provision (eg access to technology) and step‐down supports into the community (eg discharge information). The outcomes from the survey were used to frame the workshop agenda and questions (see Table 1) to ensure the workshop addressed the aim of improving consumer choice. In the current project this was a key first step. In CIPP framework terms this is understanding the ‘context’. Understanding the context can help identify any problems, understand the community needs and then consider the most appropriate response to address those needs [19].

### 3.2 Stage 2. Education Half‐Day Session (Process and Input)

Following the education session, an anonymous survey was administered by the educators to ascertain if the session had addressed the gap in knowledge which was previously identified. From the 13 attendees, 12 people provided feedback (one consumer, five employed staff and six carers). All respondents agreed or strongly agreed that the presentation and learning materials supported their understanding of codesign. A small sample of open‐ended responses is shown below:‐

- ‘Understanding the types of power, I hold and being aware of this in my practice’
- ‘Yes, the ideas of coproduction from the planning stage to codesign, co‐delivering, evaluation in future Focus group’
- ‘Listening and valuing other peoples perspectives’
- ‘This is very important … sharing training with people from mixed training, cultures and experiences. Thank you for trying to give us a voice’

The use of the education session was purposely included to address the problem of a lack of knowledge or understanding which may have directly impacted on the success of the codesign process. When considered within the CIPP framework, it indicates a potential issue or gap with the ‘process’, in particular around knowledge, which may have impacted on the success of the codesign process [18, 19]. The feedback from the education session showed it is important to reconsider the process being undertaken and provide additional inputs when needed. In this project, there was value in providing information to codesign participants before undertaking any workshops. Due to the low response rate no formal analysis was conducted on this data.

### 3.3 Stage 3. Codesign Workshops (Input)

The third stage of the project involved a series of workshops including a consumer only day. Details of the agenda, topics for each session and attendees is shown in Table 1. In codesign, the facilitation of workshops tends to be considered as a key element in the generation of ideas and potential solutions [3]. In terms of the CIPP framework this aspect might also be considered as a critical component [19]. For this project, ‘input’ was provided to participants to inform them about the survey results and, in exchange, participants provided input and ideas which built upon the initial results to expand and deepen understanding around the project needs. The input component of the CIPP framework is considered as being a means to assess the programme/projects proposed strategy to be responsive to the needs of the beneficiaries or stakeholders identified. Some of the recommendations in response to the question in workshop 3 “what do we need to consider to expand on offering more diversity of choice?” can be found in Appendix C. The result of the formal evaluation examining the outcomes for consumers pre and postimplementation of the new outcomes from the codesign process is reported elsewhere. The outcomes examined items such as perceptions of choice, recovery and satisfaction.

### 3.4 Stage 4. Postworkshop Feedback (Process)

The postworkshop survey, found that all participants (100%) highly agreed that they were *seen*, *understood* and *had a voice* in the codesign workshops. Seventy‐eight percent (seven people) highly agreed they were heard with 22% (two people) feeling moderately agreeing they felt heard. The qualitative findings indicate that there were four themes along with recommendations.

#### 3.4.1 Feeling Heard and Seen

Participants reported feelings of validation and acknowledgement. As one participant noted, “e*veryone had a say. No one was rushed or made to feel unimportant*”. The response emphasises the respectful atmosphere fostered by facilitators. The approach created a sense of shared respect and mutual understanding amongst participants. Another attendee commented that “*participants listened and were listened to with respect*”. However, in contrast, one attendee commented that “*not all were ‘*heard’ or ‘seen’” as such addressing the diverse needs and encouraging equitable participation is vital in future considerations.

#### 3.4.2 Impact of the Process Change and Hope

The codesign process instilled a sense of optimism in participants. Attendees generally expressed appreciation for the opportunity to participate, “*thank you for letting me be involved. I feel more connected and hopeful*”. The sentiment was shared by others in terms of connection and hope. Participants believed that their involvement might result in improvements in the healthcare system, [I] *“hope to see change”*. For many, the workshops provided an opportunity to engage in meaningful dialogue and conjure/imagine a different future through positive change “*a lot of good ideas put forward*”. In addition, the workshops sought to encourage attendees to challenge their own biases/assumptions and learn from the experiences of others, as one participant commented, *“you are making me think*”.

#### 3.4.3 Lack of Knowledge

Despite the positive environment, some participants expressed a desire for clearer and more accessible information. One participant shared a sense of uncertainty, “*I still feel ignorant regarding the occupational and clinical therapies available on wards*” indicating that whilst the workshops were informative it was clear participants would have benefited from more information about the services already available. Additionally, more “*information from staff re their constraints/needs/wishes*” was raised, especially due to the challenges posed by the COVID19 pandemic.

#### 3.4.4 Areas for Improvement

Participants highlighted specific areas for improvement. Some felt that additional workshops would allow facilitators to do more in the time allotted and to prepare to enhance the overall quality and depth of the sessions. As one participant suggested, “*increase workshops to enable facilitators more time and support to organise the many tasks involved*”. Timing was another concern for some, as some carers struggled to attend sessions scheduled during working hours. The running of the sessions could be better considered in light of understanding carers working lives. In terms of the CIPP framework this stage is considered as the ‘process’ which is intended to provide an ongoing check so adaptation can be made in an iterative manner. It offers the opportunity to document the process, establish if the process is achieving what was intended and provides an opportunity for feedback [18].

### 3.5 Stage 5. Project Staff Reflections (Product/Sustainability)

Personal reflections of the four team members were documented by the project lead and consumer academic during multiple debrief sessions after each workshop and operations meetings with the broader project implementation team. The reflections have been aggregated to ensure anonymity. Overall, we agreed the codesign process was peppered with challenges around three main themes, engagement and adaptability, time and expectations and power. Despite these challenges we also understood we needed to be innovative and adaptive to achieve success especially in light of COVID‐19 restrictions. We reflected that one of the key aspects of the project was the focus and incorporation of the consumer voice. The need for greater consumer involvement was a recommendation from the Victorian Royal Commission into Mental Health [21]. However, it was evident in the early stages of the project that the project requirements and what was achievable were at odds with each other, in particular around time.

#### 3.5.1 Engagement and Adaptability

We found that consumer engagement was challenging due to the health challenges many faced during the codesign period. We partially attributed this to the fact many had only recently been discharged from the acute inpatient setting. The initial recruitment undertaken involved the project lead directly contacting each consumer, however, we found this did not prove to be a good strategy. As a result, we chose to undertake a new strategy which involved a consumer peer worker, who the consumer had prior contact with, making a soft introduction to the project staff. Once the connection was made the project staff were able to engage in conversation and provide information about the codesign process. This approach enabled us to provide consumers with support to access the codesign workshops via videoconferencing and ensure there was a follow‐up debrief phone call.

Some of the consumers were reluctant to attend the in‐person or online workshops and as a result we offered them the opportunity to provide feedback as a written response or individual conversation if they preferred. We did not anticipate, on the day of the workshops, there would be significant connectivity issues resulting in consumers attempting to join, with varying success, via their mobile phone. This could have been addressed through providing onsite facilities with access to free WIFI to overcome this challenge.

Clinical engagement was perhaps the greatest challenge we experienced throughout the codesign process. We feel this was due to the public health sector in Melbourne, Victoria, being in a code brown state of emergency [23], with many COVID‐19 cases in the community and in hospital beds. This impacted on the availability of staff participants to engage in the codesign process due to staff shortages or conflicting priorities. This was highlighted by the drop in attendance by the time we reached the final workshop.

In contrast, we found there was strong and consistent carer participant engagement with a total of 8 carers involved in the codesign. The success of this engagement might be attributed to a specific funded carer engagement programme which had been implemented at the health service for some years. The programme employed a soft, inclusive approach to engagement through participation in events and activities thereby creating an extensive network of carers. In contrast there was no funded consumer engagement programme which may have contributed to the challenges with consumer engagement.

#### 3.5.2 Time and Expectations

Aligning with prior research we found one of the key aspects to conduct the codesign was time [8, 10]. In the State of Victoria, we were in a stage of immense reform within the mental health sector following the findings from the Royal Commission into Mental Health [21] as such there were no prescribed guidelines to follow for this type of project. It was evident that there was a lack of organisational readiness to instigate timely codesign, this resulted in at least two agreed extensions to the delivery of the project. A further component impacting on time was the challenges around consumer engagement. This was highlighted in carer recruitment compared to consumer recruitment highlighting the value of successful carer engagement programme with no consumer equivalent.

In addition, the added complexity of COVID‐19 and the ‘work from home’ order for all non‐patient facing staff resulted in the process taking much longer that originally envisaged. We made the decision early that if the codesign process was to be authentic, meaningful and community oriented then we need to allow sufficient time to build relationships, so this resulted in the codesign phase being a more realistic 1‐year process. However, this decision did not align with the project timeline and hierarchical expectations. This proved to be a further challenge.

#### 3.5.3 Power

These challenges lead to our considerations around power. The project team felt they had, through various ways, for example, separate breakout rooms and an education day addressed the issue of power. However, we found evidence that the issue of power existed in both implicit (unconscious) and explicit (conscious) bias [24]. Our implicit bias was our belief we needed to balance timeline pressure with the desire to be authentic. Explicit bias and power in the codesign process we felt was due to organisational structures and perceptions of who constitutes being an expert which added a layer of complexity. We were consciously aware there was a lack of consumer and staff feedback. In addition, there was a reduction in staff attendance over time. It was difficult to ascertain if this was due to disengagement or if indeed staff faced challenges around not being released to attend the workshops. Regardless, these issues support the idea around a lack of organisational readiness. In summary, we felt it was apparent through this project that there needed to be a greater focus on individual conversations earlier in the project. The nature of the project and the various stakeholders (e.g., government agencies, public health workers, consumers and carers)) were, at times, focused on problems (either personal or organisational) rather than solutions. However, we acknowledge that these reflections only capture the project team and do not capture specific feedback or reflections from supervisors, managers or the broader stakeholder group.

The personal reflections detailed align with the final stage in the CIPP framework of *product and sustainability*. To conduct a product evaluation in CIPP terms is to ‘ascertain the extent to which the needs of all the participants were met’ (p. 66) [19]. The purpose of this stage in broader CIPP terms is to measure, interpret, and judge the outcomes based on merit, worth, significance and integrity. For this project we considered the ‘product’ as being a successful codesign process with an agreed way forward for implementation of various ideas which would improve treatment choices, satisfaction, and recovery for consumers [18]. However, when we considered these challenges with the CIPP framework (product and sustainability), it highlights a potential lack of rigour in our process or an unintended consequence. At this stage, according to the CIPP framework it is critical to understand and meet participant needs [19]. One means to address this is via project team reflections, such as the one provided, to change the process in an iterative manner thereby revisiting that process. However, this did not occur. There might be several reasons for this including differing expectations, team or organisational readiness, time pressure or external reasons beyond the project team's control such as the COVID‐19 restrictions.

## 4 Discussion

This paper describes the process undertaken in a codesign project which sought to enhance consumer choice and autonomy in an acute inpatient setting. The use of the CIPP framework, post hoc, proved helpful in providing a systematic way to understand and describe the practicalities, steps and purpose needed to undertake a codesign process within a mental health setting. This process and data collection yielded some new thinking and considerations around the use of codesign in service improvement, the involvement of stakeholders and interpretation of activities undertaken. However, the study also showed there are some distinct similarities and differences *within* this codesign process and *across* that reported in prior research. We will seek to unpack each of these.

Similar to prior research, it is evident from this project that codesign is complex and multifaceted, with both challenges and benefits [11]. Despite the efforts of the project team to create an inclusive, participatory approach to the codesign process, the team felt there were still challenges around what needed to be done, by whom and by when, in particular staff and consumer involvement. However, those participating in the workshops provided feedback that they felt both seen and heard. Although the broader benefits to codesign are well‐known, reports of positive consumer responses following a codesign workshop is scant since the focus tend to be on overcoming challenges [8]. This discrepancy, similar to the broader literature, might be attributed to the fact the project team felt feel time pressured to produce new knowledge to ensure the project meets pre‐specified deadlines [10]. This pressure can place undue stress on project staff seeking to ensure an authentic process whilst balancing expectations. However, in the current study, it could be argued that the project team had some success in meeting the expectations of the participants. In terms of the CIPP framework time and resources are two common limitations but can be overcome with effective leadership and decision‐making [25]. Aligning with the CIPP framework, this highlights the iterative nature undertaken for this codesign process enabled adjustments to be made to overcome the challenges as they occurred and could be considered as one of the key learnings.

A further challenge, commonly reported in codesign literature, is the concept of power [14]. A power imbalance can occur when the agenda and rules only involve those with decision making positions [14]. This can be exacerbated when power differentials are not addressed adequately, and consumer leadership is based on convenience rather than specific expertise [13]. Before commencing the codesign process the project team was acutely aware of the need to address any power imbalance by ensuring representation in the planning stages. Despite the current project iteratively reflecting on the process undertaken we identified issues which were not successfully addressed, such as lack of clinical staff and consumer engagement, which was attributed to a power imbalance. This iterative approach is considered a strength in CIPP framework terms since it enables the ability to monitor and reevaluate the process to address any issues [19]. However, in this study it was difficult to tease out *if* there were any underlying factors which may have driven any power imbalance or if the lack of engagement was due to other circumstances. For this project, this may be considered as a missed opportunity since we had no data to support or dispute this idea and should be a consideration for future codesign activities. It also raises the question of what constitutes ‘early identification’ with respect to addressing issues within the CIPP framework given the lengthy, complex process involved in codesign [18].

Despite these challenges, there were also benefits for those who were involved in the codesign workshops such as feeling hopeful and supported which aligns with prior findings [8]. The education half‐day, which sought to upskill attendees, was well received and a strength of this codesign process. This approach has, to the authors knowledge, not previously been reported and is a key learning. Training and education are recommended for all those involved in codesign (both with and without a lived‐experience) and is considered as a key aspect to success. However, to‐date there little is known about the impact such training may have on participant experiences and success of the codesign process. This highlights how the use of a reflective, iterative approach to the codesign process is valuable since the need for an education day was established early in the project following the initial data collection stage. Not only did the education session provide knowledge it was an opportunity for participants to share their own experiences and may have been the foundational step in empowering participants thereby removing any potential power imbalance. Ensuring a greater understanding around the process and the required inputs to achieve success are key components in the CIPP framework as such is a recommended inclusion for future projects undertaking codesign methodologies. However, future projects and studies may wish to expand on the current findings to compare any difference in participation experiences depending on whether explicit education was provided or not.

A further strength could be considered as the cofacilitation of the workshops with a different facilitator representing each different cohort and an overall facilitator for the larger group. It is known there can be enhanced engagement when cofacilitation is used within a codesign process and is another means to address any potential power imbalance [7]. However, this aspect of the workshops was not featured in the feedback from participants suggesting either the person who facilitated (and their role) was not a concern or the process ran smoothly hence this was not identified as either a benefit or challenge.

Overall, we found the current project aligned well with the CIPP framework and the key considerations needed in a codesign process. Like the codesign process, the strength of the CIPP framework lies in the iterative and monitoring nature at each stage of the process. This ensures issues can be identified and addressed early of the process [19]. However, we recommend that further work is needed to the test and refine the CIPP framework prospectively within a codesign process. If greater alignment can be made it may provide foresight thereby smoothing the process further.

## 5 Future Directions

Early and more effective communication is a key consideration for the future of codesign projects and initiatives. It was evident there were broader issues and problems beyond the scope of the project itself which needed to be expressed and heard. One method to enhance communication, address power and barriers to engagement in the future is through the provision of a more open dialogue approach, such a restorative practices approach [26]. Although this approach tends to be used in the justice and education systems following a specific issue it is also a means to actively listen to differing views, understand others' perspectives and work collaboratively towards a solution and agreed outcome [26]. Although a power imbalance was not necessarily a key feature of the current study, this approach if used in the early stages of any codesign project may go some way to address beliefs towards power thereby raising engagement of all stakeholders. In addition, future projects should consider a more balanced project team with shared ownership and responsibility among a consumer, a carer and a nonlived experience staff member. This is one way to address engagement challenges. In essence there is a need for greater collaboration, communication, and embedded support within an organisation to ensure safety, success and growth so we can truly work together to support system reform.

## 6 Conclusion

The nuance of codesign within health services means prior approaches should be used with caution since each project should be adapted to meet the specific needs of the community. Despite challenges, using codesign will continue to grow as the preferred method of ensuring services meet consumer needs. As such the CIPP framework is one means to make sense of and provide a model to guide this complex process enabling considerations around; What needs to be done? How should it be done? Is it being done? Is it succeeding? [19]. Future considerations should include the need for a formal education session and more open dialogue, such as a proactive restorative approach [26], to address stakeholder expectations and address possible power issues.

## Author Contributions

**Michelle Kehoe:** conceptualisation, investigation, writing – original draft, methodology, writing – review and edit, formal analysis, data curation, validation. **Hannah Friebel:** conceptualisation, investigation, data curation, project management. KR: **Kirsty Rosie:** data curation, conceptualisation, investigation, writing – review and editing. **Paul Kremer:** data analysis, writing – review and editing. **Frances Shawyer:** project administration, validation, writing – review and editing. **Graham Meadows:** funding acquisition, conceptualisation, methodology, validation, writing – review and editing. **Ingrid Ozols:** conceptualisation, validation, writing – review and editing.

## Ethics Statement

Ethical approval for the study was obtained from the Monash Health and Monash University Human Research Ethics Committees. All participants were older than 18 years of age and provided written or verbal consent.

## Conflicts of Interest

The authors declare no conflicts of interest.

## Figures and Tables

**Figure B1:**
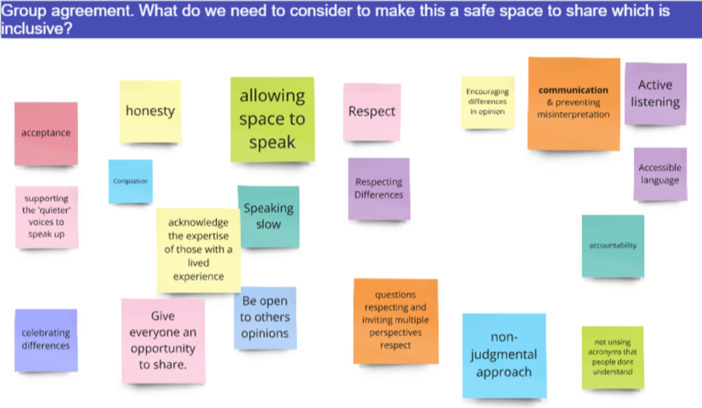
Snapshot of Miroboard showing the workshop agreement.

**Figure C1:**
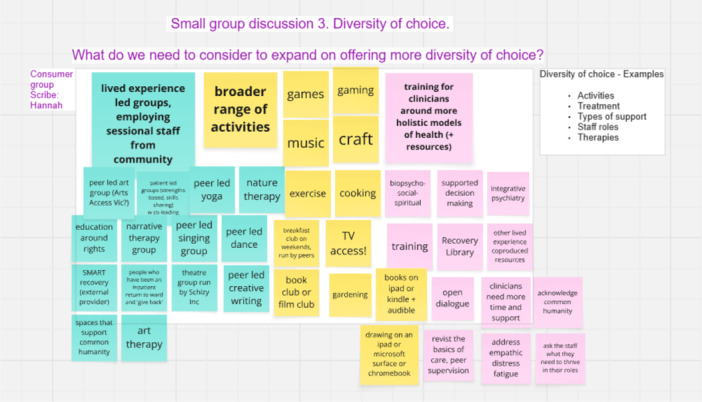
Example Miroboard activity.

## Data Availability

The data that support the findings of this study are available on request from the corresponding author. The data are not publicly available due to privacy or ethical restrictions.

## Appendix A

**Table A1:** Project stages, CIPP components and associated activities.

Project stage	CIPP component	Codesign/eval activities
1. Preworkshop survey and data collection	Context evaluation And Input evaluation	Survey stakeholders^a^ to gain broad insight into the needs of the intended beneficiaries^b^ and any possible problems for the new programme/initiative. Assess and summarise possible programme goals in light of beneficiaries' assessed needs.
2. Education day and feedback	Process evaluation And Context evaluation	Identification of possible problems and gaps which may impact on success of the codesign process. Ensure the programme's proposed strategy is responsive to assessed needs and feasibility.
3. Codesign workshops	Input evaluation	Discuss survey (input) evaluation findings in a feedback workshop. Assess the programme's proposed strategy for responsiveness to assessed needs and feasibility.
4. Post workshop feedback	Process evaluation	In collaboration with the programme's staff, maintain a record of programme events, problems, costs, and allocations
5. Project staff reflections	Sustainability (Product) evaluation	Interview programme beneficiaries to identify their judgments about what programme successes should and could be sustained.

^a^ Stakeholder were all those involved in the codesign process and included clinical staff, peer workers, consumers, carers, management and academics.

^b^ Beneficiaries are the intended recipients of the new programme which was codesign for example. Those in the acute mental health inpatient setting.
